# A novel missense variant c.G644A (p.G215E) of the *RPGR* gene in a Chinese family causes X-linked retinitis pigmentosa

**DOI:** 10.1042/BSR20192235

**Published:** 2019-10-18

**Authors:** Jiewen Fu, Jingliang Cheng, Qi Zhou, Chunli Wei, Hanchun Chen, Hongbin Lv, Junjiang Fu

**Affiliations:** 1 Key Laboratory of Epigenetics and Oncology, The Research Center for Preclinical Medicine, Southwest Medical University, Luzhou, Sichuan, China; 2 Department of Ophthalmology, The Affiliated Hospital of Southwest Medical University, Luzhou, Sichuan, China; 3 Department of Biochemistry and Molecular Biology, School of Life Sciences, Central South University, Changsha, China

**Keywords:** Expression, missense mutation, Retinitis pigmentosa, RPGR gene, Targeted next-generation sequencing (TGS)

## Abstract

The mutations in patients with X-linked retinitis pigmentosa (xlRP) have not been well described in the Chinese population. In the present study, a five-generation Chinese retinitis pigmentosa (RP) family was recruited; targeted next-generation sequencing (TGS) was used to identify causative genes and Sanger sequencing for co-segregation. RNA-seq data analysis and revere transcriptional-polymerase chain reaction (RT-PCR) were applied to investigate gene expression patterns of RP GTPase regulator (*RPGR*) in human and *Rpgr* in mouse. A novel, hemizygous, deleterious and missense variant: c.G644A (p.G215E) in the *RPGR* gene (NM_000328.2) exon 7 of X-chromosome was identified in the proband, which was co-segregated with the clinical phenotypes in this family. RNA-seq data showed that *RPGR* is ubiquitously expressed in 27 human tissues with testis in highest, but no eye tissues data. Then the expressions for *Rpgr* mRNA in mice including eye tissues were conducted and showed that *Rpgr* transcript is ubiquitously expressed very highly in retina and testis, and highly in other eye tissues including lens, sclera, and cornea; and expressed highly in the six different developmental times of retinal tissue. Ubiquitous expression in different tissues from eye and very high expression in the retina indicated that RPGR plays a vital role in eye functions, particularly in retina. In conclusion, our study is the first to indicate that the novel missense variant c.G644A (p.G215E) in the *RPGR* gene might be the disease-causing mutation in this xlRP family, expanding mutation spectrum. These findings facilitate better understanding of the molecular pathogenesis of this disease; provide new insights for genetic counseling and healthcare.

## Introduction

Retinitis pigmentosa (RP) (OMIM 268000) is a large genetic heterogeneity of inherited ocular diseases that results in a progressive retinal degeneration affecting 1 in 3000–5000 people [1–3]. Inheritance patterns in RP include autosomal recessive (arRP), autosomal dominant (adRP), and X-linked inheritance (xlRP) [4]. XlRP is a severe form of inherited retinal degeneration that primarily affects the rod photoreceptors with an early onset of night blindness and progressive reduction in the visual field, often causing patients to become legally blind by the age of 30–40 years [5,6].

Hartong et al. [7] estimated that 5–15% of RP is inherited through a model of X linkage. RP2 (OMIM 312600) is caused by mutation in the *RP2* gene (OMIM 300757). RP23 (OMIM 300424) is caused by mutation in the *OFD1* gene (OMIM 300170). Both RP3 (OMIM 300029) and RP15 (OMIM 300029) is caused by mutation in the RP GTPase regulator (*RPGR*) gene (OMIM 312610) [8–10]. The *RPGR* gene was also known as *COD1, CORDX1, CRD, orf15, PCDX, RP3, RP15*, or *XLRP3*. Inheritance of RP3 was described as X-linked recessive, while in RP15, both males and carrier females affected presented a wide spectrum of clinical features ranging from asymptomatic to severe RP. RP6 (OMIM 312612) has been mapped to chromosome Xp21.3-p21.2; RP24 (OMIM 300155), to Xq26-q27; and RP34 (OMIM 300605), to Xq28. But genes responsible for RP6, RP24 and RP34 have not been identified yet.

Mutation in the *RPGR* gene is believed to account for approximately 70% of xlRP and an estimated 11% of all RP patients [11]. In addition, *RPGR* mutations also caused syndromic RP. Dry et al. (1999) [12] identified an IVS5+1G-T splice site mutation in the *RPGR* gene in an xlRP family with recurrent respiratory infections. Furthermore, Ayyagari et al. (2002) [13] described a family in which ten males had primarily macular atrophy causing progressive loss of visual acuity with minimal peripheral visual impairment. One additional male showed extensive macular degeneration plus peripheral loss of retinal pigment epithelium and choriocapillaries. Kurata et al. (2019) [14] investigated xlRP from 12 Japanese unrelated families harboring mutations of *RPGR* or *RP2* identified 11 pathogenic mutations with 6 and 5 mutations in *RPGR* and *RP2*, respectively, suggesting the possibility that *RP2* mutations are relatively highly prevalent in Japanese.

Although mutations in the *RPGR* gene caused xlRP of Western European ancestry and Japan, *RPGR* with xlRP and genotype–phenotype correlations in the Chinese population have not been well described. Here, we applied targeted next-generation sequencing (TGS) technology to identify a novel, missense mutation of *RPGR* gene in a Chinese family with xlRP, expanding its spectrum of mutations.

## Materials and methods

### Pedigree, clinical assessment, sample collection, and DNA extraction; and ethical statement

**Figure 1:**
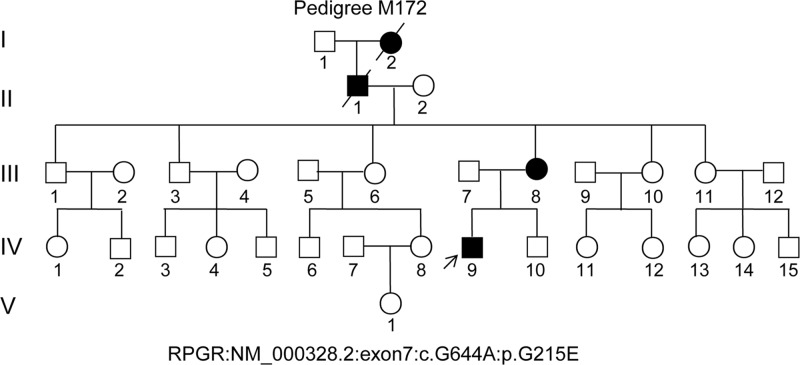
M172 pedigree with xlRP in the proband (IV:9) Family numbers and disease-causing mutations are presented. Normal individuals are shown as clear circles (females) and squares (males), the affected individual is shown as filled symbol (circles for females and squares for males). The patient above the arrow indicates as a proband (IV: 9), with the hemizygous, missense variant of the *RPGR* gene: NM_000328.2:c.G644A (p.G215E).

This pedigree consisted of a proband (Figure 1, pedigree IV: 9, arrow), five generations and 32-related family members (Figure 1). For clinical diagnosis, a detailed clinical history and ophthalmic examinations were performed in proband, as described in previous studies [4,15]. Fresh peripheral blood was taken and human genomic DNA (gDNA) was isolated using our standard phenol/chloroform method from blood leukocytes of the proband and pedigree members who were accessible [16,17]. Blood samples from 100 RP-unrelated, ethnically matched, and healthy control volunteers were collected. The research had been carried out in accordance with the World Medical Association Declaration of Helsinki, Ethical Committees approval by the Southwest Medical University, and written informed consent was obtained from all subjects.

### TGS

The panels of 195 genes for TGS analyses on the DNA sample from the proband (M172) were designed in the Illumina paired-end libraries [18,19]. The capture Agilent probes were used in previously published studies [18–20]. Isolated proband gDNA was randomly sonicated into 300–500 bp fragments, phosphorylated, hybridized, and sequenced on Illumina HiSeq 2000 following the manufacturer’s protocols [20,21]. Then, paired-end sequencing Illumina reads were aligned to the human hg19 reference genome. SNPs and INDELs variations were refined using a toolkit Atlas-SNP2 and Atlas-Indel2 (GATK version 1.0.5974) [22]. The pathogenic variants in all candidate genes were applied to online control databases, CHARGE consortium, 1000 Genome Project, ANNOVAR, ESP-6500, and Exome Aggregation Consortium (ExAC) databases [23].

### Primer design, PCR amplification, and Sanger sequencing

For putative mutation verification and co-segregation analysis, PCR amplification and Sanger sequencing of variant was applied to gDNA of all the available individuals [21,24]. Online Primer 3 (http://primer3.ut.ee/) was used to design the primers at least 50  bp upstream and downstream from the mutation. Primer pair (RPGR-M172) was designed by gDNA sequences containing identified *RPGR* mutation: NM_000328:exon7:c.G644A (Table 1). A product with 544 bp was amplified using gDNA as the template. Then, the PCR products were sequenced on an ABI-3500DX sequencer through the specific primer RPGR-M172L in Table 1. Unrelated controls were sequenced using aforementioned primers of RPGR-M172 (L+R).

**Table 1:** The sequences of PCR primers and PCR product sizes

Primer name	Left primer	Sequence (5′–3′)	Right primer	Sequence (5′–3′)	Size	°C
RPGR-M172	RPGR-M172L	Acactgctaggttttgggga	RPGR-M172R	Gaacgcagggaacagaacag	544	60
RT-rpgr	RT-rpgr-nL	Gcagcaccttaggctcaatc	RT-rpgr-nR	Aggtgtggttccttccacag	374	60
RT-b-actin-m	RT-b-actin-mL	tgttaccaactgggacgaca	RT-b-actin-mL	tctcagctgtggtggtgaag	392	60

### Protein structure and bioinformatics analysis

The functional classification of proteins and comparison in different species for RPGR was performed through an online NCBI program (https://www.ncbi.nlm.nih.gov/homologene?Db=homologene&Cmd=Retrieve&list_uids=55455) [15,25].

The *RPGR* mRNA expression profiles in human normal tissue samples from 95 human individuals representing 27 different tissues were also obtained by RNA-sequencing to determine tissue specificity through an online NCBI database (https://www.ncbi.nlm.nih.gov/gene/6103/?report=expression) [26].

### RNA isolation and revere transcriptional-polymerase chain reaction

RNA isolation from mice tissues and semi-quantitative revere transcriptional-polymerase chain reaction (RT-PCR) was performed according to our previously reported standard protocols [4,24]; the *β-actin* gene of mouse served as an internal control. RT-PCR primer pair, RT-rpgr, targeting the mouse *Rpgr* gene (GenBank No.: NM_001177950.1) which spanned three introns with 374 bp, was also designed and synthesized (Table 1). Primer pair RT-b-actin-m for mouse *β-actin* gene with 392 bp was described previously [4]. We performed PCR amplification for the *Rpgr* gene with 30 cycles and the β*-actin* gene with 25 cycles, respectively. Each assay was performed thrice.

## Results

### Pedigree recruitment and clinical characteristics

**Figure 2:**
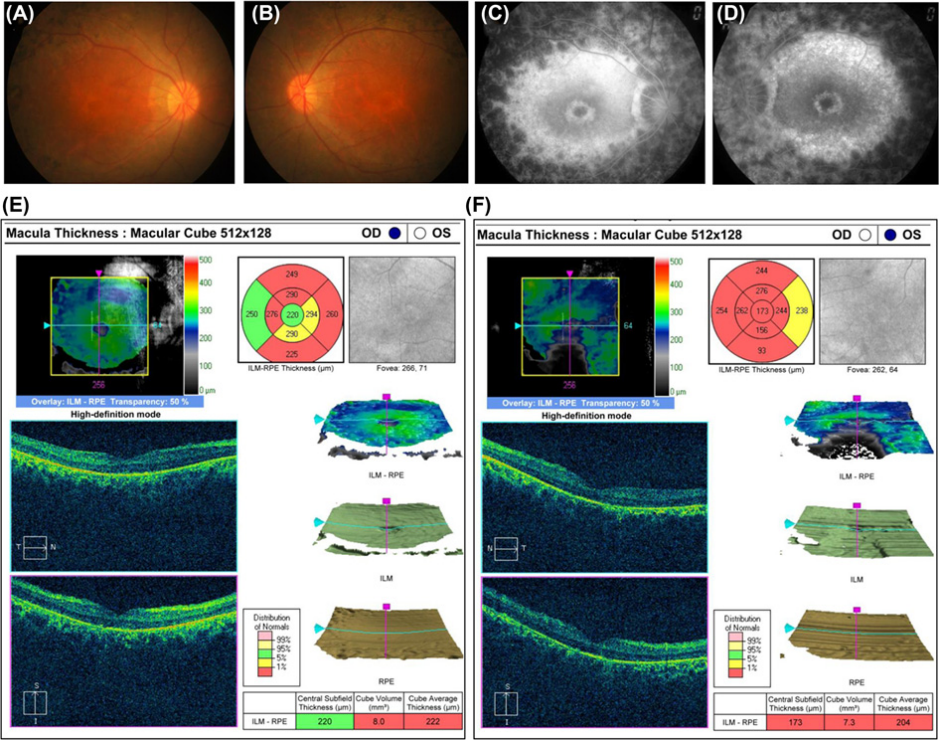
Representative FP and SD-OCT of proband (**A,B**) Color FP of proband (right and left, respectively)*.* (**C,D**). FFP of proband (right and left, respectively). SD-OCT of proband macula for quadrant measuring retinal thickness (between the inner limiting membrane and the retinal pigment epithelium: ILM-RPE) at the right eye (OD, (**E**)) and the left eye (OS, (**F**)). Top right: Quadrant measurements of retinal thickness in the eye (between the inner limiting membrane and the retinal pigment epithelium: ILM-RPE). Note the thickness reductions in the macula. Bottom right: The average macular thickness. These are represented in colors that correspond to the normal distribution of macular thickness values. Note that the average macular thickness (cube average thickness) is indicated in the bottom right chart (as well as all of the macular quadrant thicknesses) are represented in red (red denotes values <1% of what would be expected compared with an age-matched reference population), indicating a significant reduction in average macular thickness in both eyes. Abbreviations: ILM, inner limiting membrane; OS, outer segment.

The proband (Figure 1, IV: 9) was a 39-year-old Chinese male with clinical signs of progression of blindness characteristic of RP. The fundus photographs (FP) and fundus fluorescent photographs (FFP) of the proband in both eyes are shown in the Figure 2. The images of FP displayed attenuated vessels, retinal pigment epithelium (RPE) atrophy (Figure 2,A,B). FFP results showed a hyperautofluorescent ring surrounding a central area of hypoautofluorescence and an atrophic macular region (Figure 2,C,D). Spectral domain-optical coherence tomography (SD-OCT) of proband macula showed a significant reduction in average macular thickness in both eyes (Figure 2,E,F). As a result, the proband in our study was presented with typical RP. The family included 32 members and five generations, all others, except his mother, maternal grandfather, and great grandmother showed similar symptoms, were normal (Figure 1). The pedigree had no consanguineous marriage history based on their genetic and pedigree analyses.

### Next-generation sequencing analysis and putative pathogenic mutation screening

Targeted capture high-throughput sequencing of RP-related genes was performed successfully using a capture panel on the gDNA sample of proband (Figure 1, pedigree IV: 9). The causative mutations were identified by automatic variant calling, filtering, and annotation pipeline in the capture sequencing data, and a single nucleotide hemizygous, missense variant (c.G644A) of exon 7 in the *RPGR* gene (GenBank No.: NM_000328.2) in the proband was identified, leading to an amino acid change from Glycine (Gly, G) to Glutamic acid (Glu, E) at codon 215 of the RPGR protein (p.G215E) (NP_000319.1) (Figure 1 IV: 9, Supplementary Table S1, highlighted in yellow). The deleterious and pathogenic aspect of c.G644A (p.G215E) mutation in the *RPGR* gene is shown in Table 2. PolyPhen-2 analysis showed probable damage for this change with score 1; MutationTaster revealed the change to be disease causing with score 1; SIFT was deleterious with score 0, which predicted to affect protein function; and I-Mutant2.0 for the free energy change value indicated decrease in stability (DDG = −0.30 kcal/mol, <0). Thus, this missense variant in the *RPGR* gene: c.G644A (p.G215E) was pathogenic in this Chinese family. This variant c.G644A (p.G215E) was searched in the ExAC and HGMD databases and found as a novel mutation (Table 2).

**Table 2:** Characteristics of RPGR variant and analysis of disease-causing effects of proband

Gene	Exon	Variation				PolyPhen-2	Mutation Taster	I-Mutant2.0	SIFT	ExAC
		Nucleotide*	Protein*	Type	Status					
RPGR	7	c.G644A	p.G215E	Missense	Hemi	PD (1)	DC (1)	DS(-0.30)	D(0)	Novel

Abbreviations: c, variation at cDNA level; D, deleterious; DC, disease causing; DS, decrease stability; G215E, Glycine (Gly) was substituted by conserved Glutamic acid (Glu) at codon 215; Hemi, hemizygote; p, variation at protein level; PD, probably damaging.

* All nucleotides and amino acids are abbreviated according to the International Union of Pure and Applied Chemistry (IUPAC).

### Mutation verification of c.G644A (p.G215E) in RPGR and segregation analysis

**Figure 3:**
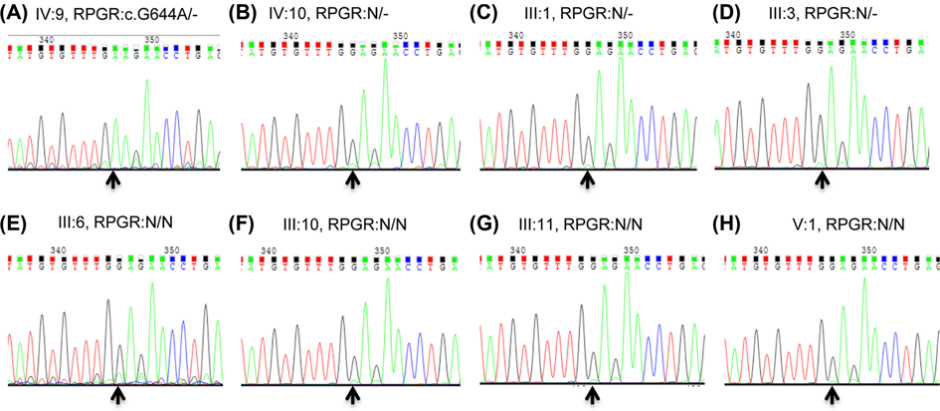
Photogram profiles for validation and segregation by Sanger sequencing (**A**–**H**) Indicate the sequencing results in IV: 9 (mutant hemizygous type), IV: 10 (wild hemizygous type), III:1 (wild hemizygous type), III:3 (wild hemizygous type), III: 6 (wild homozygous type), III: 10 (wild homozygous type), III: 11 (wild homozygous type), and V:1 (wild homozygous type), respectively. The arrows indicate mutation at the nucleotide position NM_001034853.1: c.G644A in the *RPGR* gene. ‘N’ indicates the wild-type of *RPGR* allele.

Albeit deficient, the Sanger sequencing was exploited to confirm the *RPGR* mutant hemizygous type of c.G644A in proband (pedigree IV: 9; Figure 3A), and to identify mutant heterozygous type in proband’s mother with RP disease (data not shown), wild-types in other family members with normal phenotypes (Figure 3B–H). Thus, this c.G644A (p.G215E) in *RPGR* was co-segregated with the disease phenotype in all tested family’s members. This mutant was absent from 100 unrelated, normal, ethnically matched controls (data not shown). The proband’s grandfather might have carried the same c.G644A hemizygous type, and great grandmother might have also carried the same c.G644A heterozygous type due to the RP phenotype, but no DNA samples were available because of death. Altogether, these findings showed complete co-segregation in the pedigree for the retinal dystrophy family and pinpoint its role in xlRP pathogenesis.

### Functional effects of variant c.G644A (p.G215E) in RPGR

**Figure 4:**
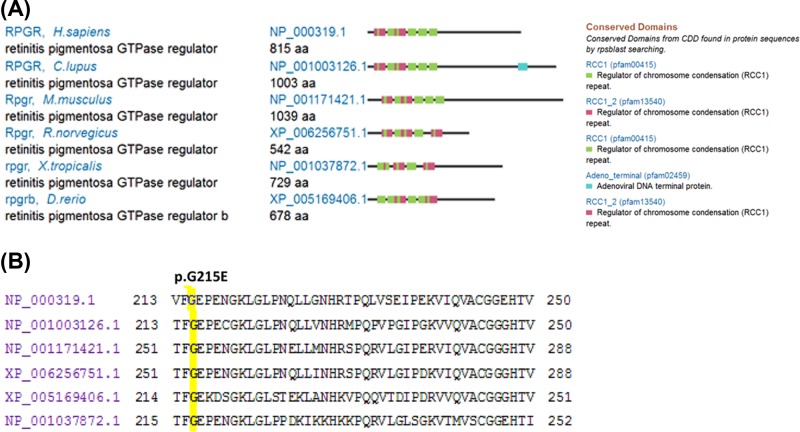
RPGR comparison and conserved domains (**A**) RPGR comparison and domains. (**B**) Conserved variant p.G215 in different species.

RPGR conservation and position for p.G215E are shown in Figure 4. By orthologous comparison of *Homo sapiens* PRGR with five other species, including *Canis lupus, Mus musculus, Rattus norvegicus, Xenopus tropicalis*, and *Danio rerio*, we found that this protein is highly conserved (Figure 4A), as well as the amino acid Glycine (G) (Figure 4B). Altogether, our study revealed that the *RPGR* hemizygous variant, c.G644A (p.G215E), might cause xlRP disease in this proband.

### Expression profiles of *RPGR* and *Rpgr* mRNA

**Figure 5:**
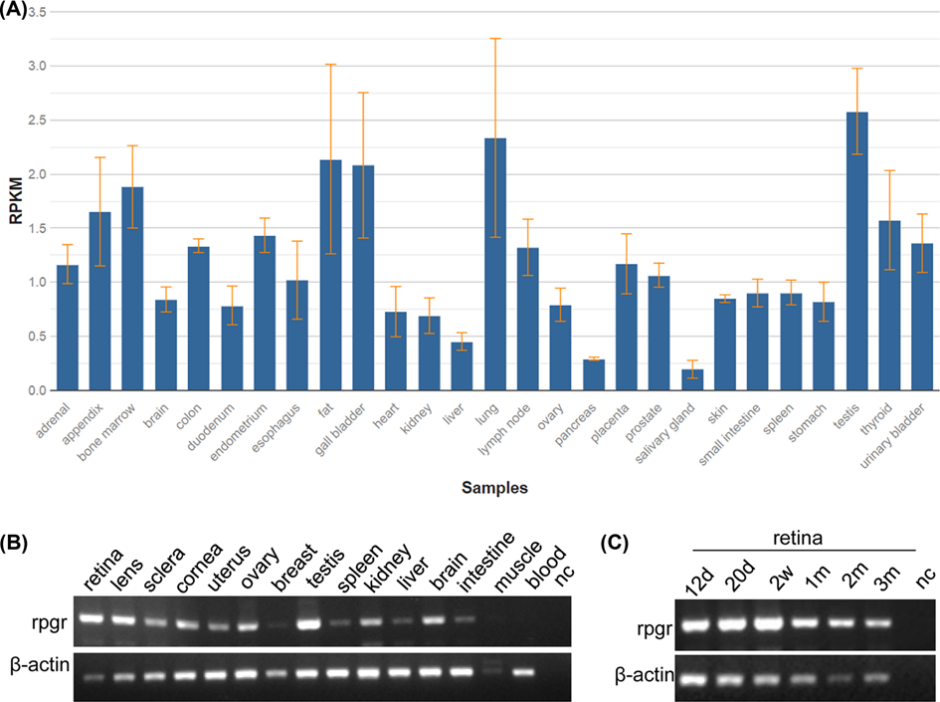
*RPGR* and *Rpgr* mRNA expression profiles (**A**) Expression profiles for *RPGR* mRNA in 27 human tissues. Expression profiles for *Rpgr* mRNA in 15 mice tissues (**B**) and in mouse 6 different development stages or times of the retinal tissue (**C**). Abbreviations: d, day(s); m, month(s); muscle, skeletal muscle; nc, negative control without any template cDNA; w, week(s). Whole eye balls at 12.5 days (12d) and 20.5 days (20d) from embryos in panel (C), respectively.

RNA-seq data showed that *RPGR* is ubiquitously expressed in representing 27 different human tissues with testis in highest (reads per kilobase million (RPKM) value: 2.58 ± 0.397) and salivary gland in lowest (RPKM value: 0.196 ± 0.082) (Figure 5A and Table 3). However, no eye tissues data were shown by RNA-seq; then the expressions for *Rpgr* mRNA in 15 different tissues and 6 different development stages of retina were conducted in mice. The results showed that *Rpgr* transcript is ubiquitously expressed very highly in retina and testis, as well as highly in other eye tissues including lens, sclera, cornea (Figure 5B); and expressed highly in the six different developmental times of retinal tissue (Figure 5C). Ubiquitous expression in different tissues from eye and very high expression in the retina indicated that RPGR plays a vital role in eye functions, particularly in retina.

**Table 3:** Expression of RPGR mRNA in human different tissues

Sample	Numbers	RPKM values	Counts
Adrenal	3	1.167 ± 0.18	82686
Appendix	3	1.653 ± 0.501	101237
Bone marrow	4	1.882 ± 0.38	319275
Brain	3	0.841 ± 0.116	70353
Colon	5	1.338 ± 0.063	246618
Duodenum	2	0.787 ± 0.179	33526
Endometrium	3	1.435 ± 0.159	118604
Esophagus	3	1.02 ± 0.361	122685
Fat	3	2.139 ± 0.876	152122
Gall bladder	3	2.081 ± 0.672	236257
Heart	4	0.729 ± 0.232	121155
Kidney	4	0.692 ± 0.164	55366
Liver	3	0.453 ± 0.082	36753
Lung	5	2.335 ± 0.918	297544
Lymph node	5	1.324 ± 0.26	264618
Ovary	2	0.792 ± 0.153	73105
Pancreas	2	0.294 ± 0.014	25662
Placenta	4	1.171 ± 0.277	191905
Prostate	4	1.066 ± 0.111	98785
Salivary gland	3	0.196 ± 0.082	29108
Skin	3	0.849 ± 0.035	107607
Small intestine	4	0.9 ± 0.128	91928
Spleen	4	0.906 ± 0.114	120628
Stomach	3	0.82 ± 0.182	79825
Testis	7	2.58 ± 0.397	789604
Thyroid	4	1.575 ± 0.458	272805
Urinary bladder	2	1.361 ± 0.27	103527

## Discussion

In the present study, we identified a hemizygous, missense variant c.G644A:p.G215E of the *RPGR* gene in the proband of a Chinese family, which led to xlRP. By searching the Human Gene Mutation Database (http://www.hgmd.cf.ac.uk/ac/gene.php?gene=RPGR) (access date, 30 August 2019), 169 pathogenic variants have been reported, including missense/nonsense (68), splicing (36), small deletions (44), small insertions (9), small indels (2), gross deletions (9), and complex rearrangement (1). It showed that different *RPGR* mutations caused different clinical correlations of diseases/phonotypes (Table 4). The proband’s mother presented typical RP symptoms in our studies, demonstrating high penetration or likely X-linked dominant. As an X-linked disease, among female carriers from 45 families by retrospective medical records review, Comander et al. (2015) [27] found that those with RPGR ORF15 mutations tended to have worse visual function than those with RPGR exon 1 through 14 mutations [28], demonstrating disease symptoms in the carriers. To the best of our knowledge, *RPGR* variant c.G644A (p.G215E) is a novel mutation, extending its spectrums of mutations. Thus, these finding shows that the *RPGR* mutation, c.G644A (p.G215E), likely causes xlRP disease in our studied Chinese pedigree.

**Table 4:** RPGR mutations and disease relations

Disease/phenotype	Number of mutations
RP, X-linked	157
RP, X-linked ?	10
Leber congenital amaurosis	1
Retinal dystrophy	1

Inheritance of RP3 was described as X-linked recessive, while in RP15, affected males and carrier females. With reference to the X-linked dominance of RP15 they stated that ‘since all females with the proposed disease-causing gene are affected, the disease is ‘dominant’ in the traditional sense of the word,’ but they agreed that the terms ‘dominant’ and ‘recessive’ can be misleading, so we called here as xlRP in our studied family.

RPGR plays a vital role as a scaffold protein in the regulation of protein trafficking, thus the cargoes can be transported to the outer segments (OSs) of photoreceptors. This trafficking process is controlled by intraflagellar transport complexes and regulated by the RPGR protein complex [29]. The C-terminus of RPGR that contains prenylated site can interact with PDE6δ, INPP5E, and RPGRIP1L, thus regulates ciliary localization of INPP5E [30,31]. Missense variations of RPGR disrupted those endogenous protein interactions which might be the common feature of RPGR causing xlRP [32]. Our missense variant c.G644A (p.G215E) of RPGR might disrupt complex formation in this family. But further study should perform to validate the hypothesis in the future. Tauroursodeoxycholic acid (TUDCA) has demonstrated therapeutic potential for RPGR patients by suppressing microglial activation and inflammation and preventing photoreceptor degeneration in Rpgr conditional knockout mice [29]. Adeno-associated viral (AAV) vectors were conducted for *RPGR* gene therapy by targeting gene expression to both rods and cones in non-human primates [33–35].

In conclusion, our study is the first to identify that the hemizygous missense variant c.G644A (p.G215E) of the *RPGR* gene in our Chinese proband, which is most likely the disease-causing mutation for xlRP, thereby expanding its spectrum of mutations. These findings facilitate better understanding of the molecular pathogenesis of this disease; provide new insights for genetic counseling and healthcare.

## Supplementary Material

Supplementary Table S1

## Abbreviations

ExAC: Exome Aggregation Consortium
FFP: fundus fluorescent photograph
FP: fundus photograph
gDNA: genomic DNA
HGMD: The Human Gene Mutation Database
INDEL: Insertion and deletion
OMIM: Online Mendelian Inheritance in Man
RP: retinitis pigmentosa
RPGR: RP GTPase regulator
RPKM: reads per kilobase million
RT-PCR: revere transcriptional-polymerase chain reaction
SIFT: Sorting Intolerant from Tolerant
SNP: Single Nucleotide Polymorphism
TGS: targeted next-generation sequencing
xIRP: X-linked RP

## References

[1] Ali M.U., Rahman M.S.U., Cao J.and Yuan P.X. (2017) Genetic characterization and disease mechanism of retinitis pigmentosa; current scenario. 3 Biotech 7, 251 10.1007/s13205-017-0878-3 28721681 PMC5515732

[2] Maubaret C.and Hamel C. (2005) Genetics of retinitis pigmentosa: metabolic classification and phenotype/genotype correlations. J. Fr. Ophtalmol. 28, 71–92 10.1016/S0181-5512(05)81029-0 15767903

[3] Ferrari S., Di Iorio E., Barbaro V., Ponzin D., Sorrentino F.S.and Parmeggiani F. (2011) Retinitis pigmentosa: genes and disease mechanisms. Curr. Genomics 12, 238–249 10.2174/138920211795860107 22131869 PMC3131731

[4] Fu J., Ma L., Cheng J., Yang L., Wei C., Fu S. et al. (2018) A novel, homozygous nonsense variant of the CDHR1 gene in a Chinese family causes autosomal recessive retinal dystrophy by NGS-based genetic diagnosis. J. Cell. Mol. Med. 22, 5662–5669 10.1111/jcmm.13841 30160356 PMC6201214

[5] Demirci F.Y., Rigatti B.W., Mah T.S.and Gorin M.B. (2006) A novel RPGR exon ORF15 mutation in a family with X-linked retinitis pigmentosa and Coats’-like exudative vasculopathy. Am. J. Ophthalmol. 141, 208–210 10.1016/j.ajo.2005.07.077 16387007

[6] Buraczynska M., Wu W., Fujita R., Buraczynska K., Phelps E., Andreasson S. et al. (1997) Spectrum of mutations in the RPGR gene that are identified in 20% of families with X-linked retinitis pigmentosa. Am. J. Hum. Genet. 61, 1287–1292 10.1086/301646 9399904 PMC1716085

[7] Hartong D.T., Berson E.L.and Dryja T.P. (2006) Retinitis pigmentosa. Lancet 368, 1795–1809 10.1016/S0140-6736(06)69740-7 17113430

[8] Roepman R., van Duijnhoven G., Rosenberg T., Pinckers A.J., Bleeker-Wagemakers L.M., Bergen A.A. et al. (1996) Positional cloning of the gene for X-linked retinitis pigmentosa 3: homology with the guanine-nucleotide-exchange factor RCC1. Hum. Mol. Genet. 5, 1035–1041 10.1093/hmg/5.7.1035 8817343

[9] Fujita R., Buraczynska M., Gieser L., Wu W., Forsythe P., Abrahamson M. et al. (1997) Analysis of the RPGR gene in 11 pedigrees with the retinitis pigmentosa type 3 genotype: paucity of mutations in the coding region but splice defects in two families. Am. J. Hum. Genet. 61, 571–580 10.1086/515523 9326322 PMC1715956

[10] Meindl A., Dry K., Herrmann K., Manson F., Ciccodicola A., Edgar A. et al. (1996) A gene (RPGR) with homology to the RCC1 guanine nucleotide exchange factor is mutated in X-linked retinitis pigmentosa (RP3). Nat. Genet. 13, 35–42 10.1038/ng0596-35 8673101

[11] Vervoort R., Lennon A., Bird A.C., Tulloch B., Axton R., Miano M.G. et al. (2000) Mutational hot spot within a new RPGR exon in X-linked retinitis pigmentosa. Nat. Genet. 25, 462–466 10.1038/78182 10932196

[12] Dry K.L., Manson F.D., Lennon A., Bergen A.A., Van Dorp D.B.and Wright A.F. (1999) Identification of a 5′ splice site mutation in the RPGR gene in a family with X-linked retinitis pigmentosa (RP3). Hum. Mutat. 13, 141–145 10.1002/(SICI)1098-1004(1999)13:2<141::AID-HUMU6>3.0.CO;2-Q 10094550

[13] Ayyagari R., Demirci F.Y., Liu J., Bingham E.L., Stringham H., Kakuk L.E. et al. (2002) X-linked recessive atrophic macular degeneration from RPGR mutation. Genomics 80, 166–171 10.1006/geno.2002.6815 12160730

[14] Kurata K., Hosono K., Hayashi T., Mizobuchi K., Katagiri S., Miyamichi D. et al. (2019) X-linked retinitis pigmentosa in japan: clinical and genetic findings in male patients and female carriers. Int. J. Mol. Sci. 20, E1518 10.3390/ijms20061518 30917587 PMC6470860

[15] Imani S., Ijaz I., Shasaltaneh M.D., Fu S., Cheng J.and Fu J. (2018) Molecular genetics characterization and homology modeling of the CHM gene mutation: A study on its association with choroideremia. Mutat. Res. 775, 39–50 10.1016/j.mrrev.2018.02.001 29555028

[16] Fu S., Cheng J., Wei C., Yang L., Xiao X., Zhang D. et al. (2017) Development of diagnostic SCAR markers for genomic DNA amplifications in breast carcinoma by DNA cloning of high-GC RAMP-PCR fragments. Oncotarget 8, 43866–43877 28410206 10.18632/oncotarget.16704 PMC5546446

[17] Fu J., Li L.and Lu G. (2002) Relationship between microdeletion on Y chromosome and patients with idiopathic azoospermia and severe oligozoospermia in the Chinese. Chin. Med. J. 115, 72–75 11930664

[18] Wang F., Wang H., Tuan H.F., Nguyen D.H., Sun V., Keser V. et al. (2014) Next generation sequencing-based molecular diagnosis of retinitis pigmentosa: identification of a novel genotype-phenotype correlation and clinical refinements. Hum. Genet. 133, 331–345 10.1007/s00439-013-1381-5 24154662 PMC3945441

[19] Zhang Q., Xu M., Verriotto J.D., Li Y., Wang H., Gan L. et al. (2016) Next-generation sequencing-based molecular diagnosis of 35 Hispanic retinitis pigmentosa probands. Sci. Rep. 6, 32792 10.1038/srep32792 27596865 PMC5011706

[20] Fu Q., Xu M., Chen X., Sheng X., Yuan Z., Liu Y. et al. (2017) CEP78 is mutated in a distinct type of Usher syndrome. J. Med. Genet. 54, 190–195 10.1136/jmedgenet-2016-104166 27627988 PMC6235689

[21] Imani S., Cheng J., Mobasher-Jannat A., Wei C., Fu S., Yang L. et al. (2018) Identification of a novel RPGRIP1 mutation in an Iranian family with leber congenital amaurosis by exome sequencing. J. Cell. Mol. Med. 22, 1733–1742 10.1111/jcmm.13454 29193763 PMC5824405

[22] Challis D., Yu J., Evani U.S., Jackson A.R., Paithankar S., Coarfa C. et al. (2012) An integrative variant analysis suite for whole exome next-generation sequencing data. BMC Bioinformatics 13, 8 10.1186/1471-2105-13-8 22239737 PMC3292476

[23] Imani S., Cheng J., Fu J., Mobasher-Jannat A., Wei C., Mohazzab-Torabi S. et al. (2019) Novel splicing variant c. 208+2T>C in BBS5 segregates with Bardet-Biedl syndrome in an Iranian family by targeted exome sequencing. Biosci. Rep. 39, BSR20181544 10.1042/BSR20181544 30850397 PMC6438871

[24] Cheng J., Fu J., Zhou Q., Xiang X., Wei C., Yang L. et al. (2019) A novel splicing mutation in the PRPH2 gene causes autosomal dominant retinitis pigmentosa in a Chinese pedigree. J. Cell. Mol. Med. 23, 3776–3780 10.1111/jcmm.14278 30892800 PMC6484291

[25] Marchler-Bauer A., Bo Y., Han L., He J., Lanczycki C.J., Lu S. et al. (2017) CDD/SPARCLE: functional classification of proteins via subfamily domain architectures. Nucleic Acids Res. 45, D200–D203 10.1093/nar/gkw1129 27899674 PMC5210587

[26] Zhou B., Wei C., Khan M.A., Chen H.and Fu J. (2019) Characterization and molecular cloning of novel isoforms of human spermatogenesis associated gene SPATA3. Mol. Biol. Rep. 46, 3827–3834 10.1007/s11033-019-04825-4 31006096

[27] Comander J., Weigel-DiFranco C., Sandberg M.A.and Berson E.L. (2015) Visual function in carriers of X-linked retinitis pigmentosa. Ophthalmology 122, 1899–1906 10.1016/j.ophtha.2015.05.039 26143542 PMC4562908

[28] Sharon D., Sandberg M.A., Rabe V.W., Stillberger M., Dryja T.P.and Berson E.L. (2003) RP2 and RPGR mutations and clinical correlations in patients with X-linked retinitis pigmentosa. Am. J. Hum. Genet. 73, 1131–1146 10.1086/379379 14564670 PMC1180492

[29] Zhang X., Shahani U., Reilly J.and Shu X. (2019) Disease mechanisms and neuroprotection by tauroursodeoxycholic acid in Rpgr knockout mice. J. Cell. Physiol. 234, 18801–18812 30924157 10.1002/jcp.28519

[30] Dutta N.and Seo S. (2016) RPGR, a prenylated retinal ciliopathy protein, is targeted to cilia in a prenylation- and PDE6D-dependent manner. Biol. Open 5, 1283–1289 10.1242/bio.020461 27493202 PMC5051646

[31] Rao K.N., Zhang W., Li L., Anand M.and Khanna H. (2016) Prenylated retinal ciliopathy protein RPGR interacts with PDE6delta and regulates ciliary localization of Joubert syndrome-associated protein INPP5E. Hum. Mol. Genet. 25, 4533–4545 28172980 10.1093/hmg/ddw281 PMC6078598

[32] Zhang Q., Giacalone J.C., Searby C., Stone E.M., Tucker B.A.and Sheffield V.C. (2019) Disruption of RPGR protein interaction network is the common feature of RPGR missense variations that cause XLRP. Proc. Natl. Acad. Sci. U.S.A. 116, 1353–1360 10.1073/pnas.1817639116 30622176 PMC6347721

[33] Beltran W.A., Cideciyan A.V., Boye S.E., Ye G.J., Iwabe S., Dufour V.L. et al. (2017) Optimization of retinal gene therapy for X-linked retinitis pigmentosa due to RPGR mutations. Mol. Ther. 25, 1866–1880 10.1016/j.ymthe.2017.05.004 28566226 PMC5542804

[34] Fischer M.D., McClements M.E., Martinez-Fernandez de la Camara C., Bellingrath J.S., Dauletbekov D., Ramsden S.C. et al. (2017) Codon-optimized RPGR improves stability and efficacy of AAV8 gene therapy in two mouse models of X-linked retinitis pigmentosa. Mol. Ther. 25, 1854–1865 10.1016/j.ymthe.2017.05.005 28549772 PMC5542800

[35] Song C., Conlon T.J., Deng W.T., Coleman K.E., Zhu P., Plummer C. et al. (2018) Toxicology and pharmacology of an AAV vector expressing codon-optimized RPGR in RPGR-deficient Rd9 mice. Human Gene Ther. Clin. Dev. 29, 188–197 10.1089/humc.2018.168 30280954 PMC6421992

